# Systematic review and meta-analysis: prevalence of alcohol use among young people in eastern Africa

**DOI:** 10.1111/tmi.12267

**Published:** 2014-01-31

**Authors:** Joel M Francis, Heiner Grosskurth, John Changalucha, Saidi H Kapiga, Helen A Weiss

**Affiliations:** 1 London School of Hygiene and Tropical MedicineLondon, UK; 2 National Institute for Medical Research, Mwanza CentreMwanza, Tanzania; 3 Mwanza Intervention Trials UnitMwanza, Tanzania

**Keywords:** alcohol use, systematic review, meta-analysis, eastern Africa, problem drinking, screening questionnaires, young people, AUDIT, CAGE

## Abstract

**Objective:**

Systematic review and meta-analysis of published studies of alcohol use among young people (age 15–24 years) in eastern Africa to estimate prevalence of alcohol use and determine the extent of use of standardised screening questionnaires in alcohol studies.

**Methods:**

Five databases (MEDLINE, EMBASE, Global Health, Africa-wide, and PsycINFO) were searched for publications until 30th June 2013. Results were summarised using the guidelines on preferred reporting items for systematic reviews and meta-analyses (PRISMA) and on quality assessment using the modified quality assessment tool for systematic reviews of observational studies (QATSO). Heterogeneity was assessed using the I^2^ statistic (DerSimonian-Laird).

**Results:**

We identified 2785 potentially relevant studies, of which 56 were eligible for inclusion. Only two studies (4%) used the standardised Alcohol Use Disorder Identification Test (AUDIT) questionnaire, and six studies (13%) used the Cut down, Annoyed, Guilt, Eye opener (CAGE) questionnaire. The reported median prevalence of alcohol use was ever-use 52% [interquartile range (IQR): 20–58%], use in the last month 28% (IQR: 17–37%), use in the last year 26% (IQR: 22–32%), and problem drinking as defined by CAGE or AUDIT 15% (IQR: 3–36%). We observed high heterogeneity between studies, with the highest prevalence of ever use of alcohol among university students (82%; 95%CI: 79–85%) and female sex workers (66%; 95%CI: 58–74%). Current use was most prevalent among male sex workers (69%; 95%CI: 63–75%).

**Conclusions:**

Reported alcohol use and problem drinking were common among diverse groups of young people in eastern Africa, indicating the urgent need for alcohol-focused interventions in this population. Few studies have used standardised alcohol screening questionnaires. Epidemiological research to investigate alcohol-focused interventions in young people should aim to apply such questionnaires that should be validated for use in this population.

## Introduction

Harmful alcohol use is a significant public health problem that often begins early in adult life. Globally, an estimated 2 billion people drink alcohol and 76 million have alcohol use disorders (AUD) (WHO 2004). The mean volume of pure alcohol consumed annually by adults globally has been estimated at about 5.0 l per capita (WHO 2004). In Africa, annual consumption of pure alcohol has been estimated to range from 4.9 to 7.1 l per capita (WHO 2004), although intake may be significantly higher because much alcohol consumption is believed to remain unrecorded (WHO 2004). Alcohol use and AUD are associated with more than 60 medical conditions and injuries (WHO 2004; Rehm *et al*. 2006), and about 4% of global mortality and 5% of disability-adjusted life year's (DALYs) lost are attributed to alcohol use (Rehm *et al*. 2009). In the African region, it is estimated that about 2.4% of deaths and 2.1% of DALYs lost are attributed to alcohol use and AUD (Rehm *et al*. 2009). Adverse effects of alcohol use include increased risk of infectious diseases such as HIV/AIDS and TB, and chronic non-communicable diseases (NCD) (Makimoto & Higuchi 1999; Horn-Ross *et al*. 2004; WHO 2004; Ahmed *et al*. 2006; Chen *et al*. 2008; Chong *et al*. 2008; Brooks *et al*. 2009; Genkinger *et al*. 2009; Brandish & Sheron 2010; Kahl *et al*. 2010; Patra *et al*. 2010; Stroffolini *et al*. 2010), as well as intentional and unintentional injuries, and social problems such as domestic violence, unemployment and decreased work productivity (Gmel & Rehm 2003; Fisher *et al*. 2007; Kalichman *et al*. 2007; Rehm *et al*. 2009; Zaleski *et al*. 2010; Abbey 2011; Aldridge-Gerry *et al*. 2011).

Factors associated with alcohol use include religion, personal income, education level, peer influence, having older sexual partners, stress and relatives and friends using alcohol (Smith *et al*. 1993; Othieno & Obondo 2000; Kuntsche *et al*. 2005; Otieno & Ofulla 2009; Ndetei *et al*. 2009, 2010; Namagembe *et al*. 2010; Amemori *et al*. 2011; Atwoli *et al*. 2011). There are few data, on the patterns of use, harmful consequences of alcohol use among young people, or on the structural and individual factors that lead to the uptake and persistence of harmful alcohol use. A better understanding of the epidemiology of alcohol use among young people is therefore required to facilitate the design of effective alcohol-focused interventions in Africa in general and eastern Africa in particular.

The aim of this article is to systematically review published studies of alcohol use among young people in eastern Africa to estimate the prevalence of alcohol use and the extent of use of standardised alcohol screening questionnaires in preparation for future alcohol-focused intervention studies in this region. The specific objectives of the review were to (i) estimate the prevalence of alcohol use among specific groups of young people (15–24 years) in eastern Africa; (ii) determine the extent of use of standardised alcohol screening questionnaires [Alcohol Use Disorder Identification Test (AUDIT), Cut down, Annoyed, Guilt, Eye opener (CAGE) in identifying alcohol use and AUD in this region; (iii) assess the quality of research papers included in the review; and (iv) describe factors associated with initiation and persistence of alcohol use among young people in eastern Africa.

## Methods

### Search strategy

Five databases (MEDLINE, EMBASE, Global Health, Africawide-information, and PsycINFO) were searched for publications to 30th June 2013. We used the following key terms: (alcohol use OR alcohol abuse) AND (young people OR adolescent OR teenage OR youth) AND (Africa OR Tanzania OR Kenya OR Uganda OR Ethiopia OR Seychelles OR Rwanda OR Eritrea OR Burundi OR Somalia OR Somaliland OR Comoros OR South-Sudan). (see search details for each database in Appendix S1*)*.

Titles and abstracts of all records identified were screened independently by two authors (JMF and HAW), and consensus on potential eligibility reached. Studies were eligible if they were conducted in eastern Africa (Tanzania, Kenya, Uganda, Ethiopia, Seychelles, Rwanda, Eritrea, Burundi, Somalia, Somaliland, Comoros and South Sudan); and included prevalence of alcohol use for young people aged 15–24 years.

Guidelines on preferred reporting items for systematic reviews and meta-analyses (PRISMA) were used (Moher *et al*. 2009). There is currently an emphasis to incorporate both qualitative and quantitative evidence in the systematic reviews (Pearson 2004; Thomas *et al*. 2004). However, in this review, we focused on determining the prevalence of alcohol use, which was the main objective and therefore we did not include qualitative research papers.

### Data extraction

We used a data extraction form to collect the following information from each eligible article: (i) country; (ii) year the study was conducted; (iii) year of publication; (iv) study population (the general population, secondary school students, primary school students, female sex/bar workers, men who have sex with men, health care service attendees and university students); (v) sample size; (vi) definition of alcohol use (ever use, current use, use in the last year, problem drinking); (vii) prevalence of alcohol use and AUD (problem drinking as classified by CAGE and AUDIT); (viii) factors associated with the initiation and persistence of alcohol use; (ix) alcohol use screening questionnaires applied; and (x) complications associated with alcohol use.

A descriptive quality assessment of the final papers included in the meta-analysis was conducted using the modified quality assessment tool for systematic reviews of observational studies (QATSO) (Wong *et al*. 2008). The original QATSO tool is composed of five quality categories that include external validity (sampling strategy used), reporting (response rate and objectivity of measurement), confounding factors, bias (privacy) and a final score based on the mentioned parameters. The primary outcome for this review is prevalence of alcohol use, and the reported response rate was modified to include three categories (>80%, 60–80%, <60%). The assessment of confounding was not required, as the studies did not provide adjustable information on risk factors for alcohol use. We did also not compute the overall final QATSO score based on the five quality categories.

### Statistical analysis

We assessed the heterogeneity of prevalence estimates using the I^2^ statistic (DerSimonian-Laird) and reported the prevalence for studies in four groups: (i) ever use of alcohol; (ii) alcohol use in the last year, (iii) alcohol use in the last month (current use) and (iv) problem drinking as defined by CAGE and AUDIT (Ewing 1984; Dersimonian & Laird 1986).

Due to significant heterogeneity between studies, we estimated the median prevalence for each group. We also performed meta-regression to analyse the association between current alcohol use and gender, study setting, and quality assessment parameters (sampling strategy, response rate, interview modality and data collection tool used).

## Results

We identified 4013 published study citations from five databases, of which 1228 were duplicates. Thus, 2785 abstracts were screened for initial eligibility to identify studies conducted in eastern Africa. We identified 696 relevant abstracts of studies conducted in eastern Africa. We conducted further screening for studies reporting on alcohol use and identified 285 abstracts for full article review. Of these 285 abstracts for full article assessment, we could not access six full articles, 11 were review articles and five were conference posters.

**Figure 1:**
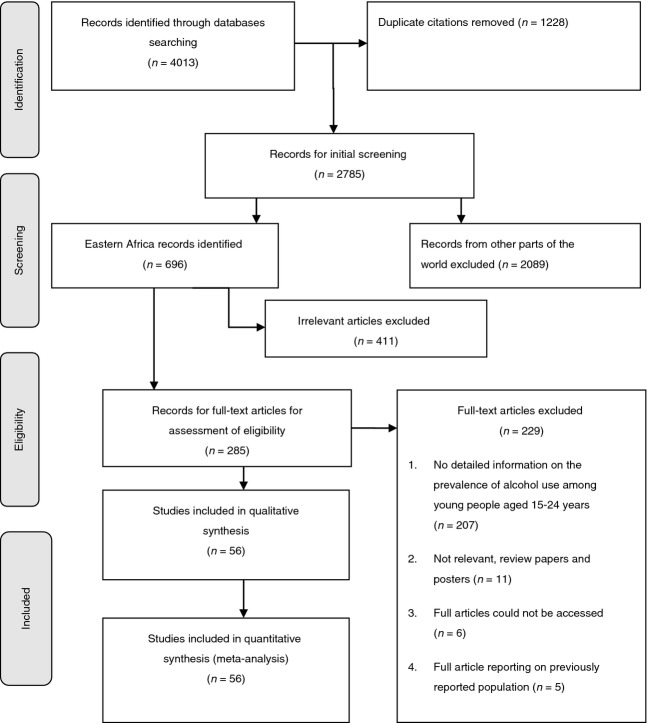
Flow Diagram for literature review.

Thus, we reviewed 263 full-text papers and identified 56 eligible for inclusion in the review. The main reason for exclusion was that the paper did not report information on alcohol use from the target population, that is, young people aged 15–24 years, or that young people were included but we could not separate the prevalence in this age group from that in older people (Figure 1).

Of the 56 eligible studies (Table 1), five reported both current alcohol use and ever use, and one study reported current use and problem drinking and are included in each of these analyses (Zein 1988; Gedif & Eshetu 2007; Mbatia *et al*. 2009; Luchters *et al*. 2011; Kagimu *et al*. 2012; Reda *et al*. 2012). The majority of studies were cross-sectional (*n* = 54, 96%), and two were case–control studies. Almost all studies (*n* = 52, 93%) were conducted in four countries: Ethiopia (*n* = 19), Kenya (*n* = 15), Tanzania (*n* = 10) and Uganda (*n* = 8); the remaining four studies were conducted in Rwanda (*n* = 2), Seychelles (*n* = 1) and Eritrea (*n* = 1). Most studies (*n* = 36, 58%) reported current alcohol use, 17 studies reported ever use of alcohol, four studies reported alcohol use in the last year, and five studies reported problem drinking. Only two studies used the AUDIT alcohol use screening questionnaire (Mbatia *et al*. 2009; Luchters *et al*. 2011), and six used the CAGE questionnaire [one conducted among female sex workers, three among the general population and two among healthcare attenders (Alem *et al*. 1999; Kebede & Alem 1999; Ghebremichael *et al*. 2009; Kullgren *et al*. 2009; Namagembe *et al*. 2010; Ao *et al*. 2011)]. Of 48 studies that recruited both sexes, only 13 studies (27%) reported gender-specific prevalence of alcohol use. Four studies reported on the factors for initiation and persistence of alcohol use (Otieno & Ofulla 2009; Ndetei *et al*. 2010; Amemori *et al*. 2011; Atwoli *et al*. 2011).

**Table 1:** Description of studies included in the systematic review and meta-analysis

First author	Year the study conducted	Country	Study population	Sample size	Prevalence	95% CI of prevalence	Alcohol screening tool	Gender	Location
Ever-used alcohol									
Taffa *et al*. (2002)	2000	Ethiopia	General population	561	15.7	12.7–18.7	None	Both	Urban
Mbatia *et al*. (2009)	2003	Tanzania	General population	275	16.7	12.3–21.1	AUDIT	Both	Urban
Zablotska *et al*. (2009)	1994–2002	Uganda	General population	3422	19.8	18.5–21.1	None	Female	Rural
Malaju and Asale (2013)	2012	Ethiopia	General population	405	31.6	27.1–36.1	None	Both	Both
Bwana (1996)	Not reported	Kenya	General population	306	54.9	49.3–60.5	None	Both	Rural
Kagimu *et al*. (2012)	2010	Uganda	General population	530	56.2	52.0–60.5	None	Both	Rural
Fekadu and Alemayehu (2009)	2008	Ethiopia	General population	634	64.0	60.3–67.8	None	Both	Rural
Ndetei *et al*. (2010)	Not reported	Kenya	Secondary school students	343	5.2	2.9–7.6	None	Both	Rural
Reda *et al*. (2012)	2010	Ethiopia	Secondary school students	1721	22.2	20.2–24.2	None	Both	Both
Kuria (1996)	Not reported	Kenya	Secondary school students	952	53.0	49.9–56.2	None	Both	Both
Otieno & Ofulla (2009)	Not reported	Kenya	Secondary school students	458	57.9	53.3–62.4	None	Both	Urban
Othieno and Obondo (2000)	1997	Kenya	Street children	50	14.0	4.4–23.6	None	Both	Urban
Atwoli *et al*. (2011)	2009	Kenya	University students	500	52.0	47.6–56.4	None	Both	Urban
Zein (1988)	1983	Ethiopia	University students	485	70.1	66.0–74.2	None	Both	Urban
Gedif and Eshetu (2007)	2006	Ethiopia	University students	674	81.6	78.7–84.5	None	Both	Urban
Twa-Twa *et al*. (2008)	2003	Uganda	Primary school students	1709	27.8	25.7–29.9	None	Both	Urban
Tegang *et al*. (2010)	2007	Kenya	Female sex workers	137	65.7	57.7–73.6	None	Female	Urban
Alcohol use in the last one year									
Rijken *et al*. (1998)	1993	Tanzania	General population	34	20.6	7.0–34.2	None	Both	Rural
Usman *et al*. (2006)	2004	Eritrea	General population	490	29.4	25.4–33.4	None	Both	Both
Mbona and Kasirye (2005)	2003	Uganda	General population	247	33.6	27.7–39.5	None	Both	Rural
Deressa and Azazh (2011)	2009	Ethiopia	University students	608	22.0	18.7–25.3	None	Both	Urban
Current alcohol use									
Maru *et al*. (2003)	Not reported	Kenya	General population	90	6.7	1.5–11.8	None	Both	Urban
Mbatia *et al*. (2009)	2003	Tanzania	General population	275	9.8	6.3–13.3	AUDIT	Both	Urban
Hargreaves *et al*. (2002)	1996	Kenya	General population	889	11	9.0–13.1	None	Both	Urban
Chande and Salum (2007)	NR	Tanzania	General population	86	11.6	4.9–18.4	None	Both	Urban
Odero and Zwi (1997)	1995	Kenya	General population	28	14.3	1.3–27.2	Breathalyser	Both	Urban
Molla *et al*. (2008)	2004	Ethiopia	General population	3044	17.9	16.5–19.3	None	Both	Both
Khasakhala and Mturi (2008)	2002	Kenya	General population	3639	19	17.7–20.3	None	Both	Both
Derege *et al*. (2005)	2001–2002	Ethiopia	General population	20434	21.3	20.7–21.9	None	Both	Both
Kitange *et al*. (1993)	Not reported	Tanzania	General population	1467	23.5	21.3–25.7	None	Both	Both
Kagimu *et al*. (2012)	2010	Uganda	General population	530	30.8	26.8–34.7	None	Both	Rural
Swahn *et al*. (2012)	2011	Uganda	General population	461	32.5	28.3–36.8	None	Both	Urban
Betre *et al*. (1997)	1994–1995	Ethiopia	General population	1436	34.3	31.9–36.8	None	Both	Urban
Mnyika *et al*. (2011)	2002	Tanzania	General population	926	34.6	31.9–37.3	None	Both	Rural
Alemu *et al*. (2007)	2003	Ethiopia	General population	628	43.8	39.9–47.7	None	Both	Urban
Boris *et al*. (2008)	2004	Rwanda	General population	539	49.2	44.9–53.4	None	Both	Rural
Tengia-Kessy *et al*. (2010)	1995	Tanzania	General population	1104	60	57.1–62.9	None	Both	Rural
Namagembe *et al*. (2010)	2006	Uganda	Healthcare service	384	16.4	12.7–20.1	CAGE	Female	Urban
Kullgren *et al*. (2009)	2007	Uganda	Healthcare service	76	21.1	11.9–30.2	CAGE	Both	Urban
Hassan *et al*. (2005)	1999	Kenya	Healthcare service	45	24.4	11.9–37.0	None	Both	Urban
Ayuku and Odero (2002)	1995–1996	Kenya	Healthcare service	778	26.5	23.4–29.6	Breathalyser	Both	Both
Luchters *et al*. (2011)	2008	Kenya	Male sex workers	222	68.9	62.8–75.0	AUDIT	Male	Urban
Kebede and Ketsela (1993)	1989–1990	Ethiopia	Secondary school students	519	9.2	6.8–11.7	None	Both	Urban
Dhadphale *et al*. (1982)	Not reported	Kenya	Secondary school students	2918	10.3	9.2–11.4	None	Both	Both
Reda *et al*. (2012)	2010	Ethiopia	Secondary school students	1721	10.4	9.0–11.8	None	Both	Both
Shiferaw *et al*. (2011)	2009	Ethiopia	Secondary school students	240	26.7	21.1–32.3	None	Both	Rural
Tengia-Kessy *et al*. (2010)	2008	Tanzania	Secondary school students	400	39.0	34.2–43.8	None	Both	Urban
Van Decraen *et al*. (2012)	Not reported	Rwanda	Secondary school students	285	43.9	38.1–49.6	None	Both	Rural
Lioul and Jemal (2009)	2005	Ethiopia	Secondary school students	810	51.5	48.0–54.9	None	Both	Urban
Faeh *et al*. (2006)	Not reported	Seychelles	Secondary school students	390	60.5	55.7–65.4	None	Both	Urban
Arnold *et al*. (2008)	2006	Ethiopia	University students	1330	19.2	17.1–21.3	None	Female	Urban
Regassa and Kedir (2011)	2010	Ethiopia	University students	606	29.7	26.1–33.3	None	Both	Urban
Amemori *et al*. (2011)	2006	Tanzania	University students	66	30.3	19.2–41.4	None	Both	Urban
Zein (1988)	1983	Ethiopia	University students	485	31.1	27.0–35.3	None	Both	Urban
Philpart *et al*. (2009)	2006	Ethiopia	University students	1378	31.2	28.8–33.7	None	Male	Urban
Gedif and Eshetu (2007)	2006	Ethiopia	University students	674	31.2	27.7–34.7	None	Both	Urban
Agardh *et al*. (2011)	2005	Uganda	University students	980	41.1	38.0–44.2	None	Both	Rural
Problem drinking									
Kebede and Alem (1999)	1994	Ethiopia	General population	4586	1.2	0.9–1.5	CAGE	Both	Urban
Alem *et al*. (1999)	Not reported	Ethiopia	General population	2997	2.6	2.0–3.2	CAGE	Both	Rural
Ghebremichael *et al*. (2009)	2002–2003	Tanzania	General population	214	15.0	10.2–19.7	CAGE	Female	Urban
Ao *et al*. (2011)	2002–2006	Tanzania	Female bar workers	723	36.2	32.7–39.7	CAGE	Female	Urban
Luchters *et al*. (2011)	2008	Kenya	Male sex workers	222	46.8	40.0–53.0	AUDIT	Male	Urban

AUDIT, Alcohol Use Disorder Identification Test.

In general, studies were of high quality (Table 2). Most (*n* = 42, 75%) used probability-based sampling and had a response rate above 80% (*n* = 33; 58.9%). However, 17 studies did not report the response rate (Bwana 1996; Kuria 1996; Odero & Zwi 1997; Othieno & Obondo 2000; Taffa *et al*. 2002; Maru *et al*. 2003; Ayuku & Odero 2002; Hassan *et al*. 2005; Mbona & Kasirye 2005; Chande & Salum 2007; Khasakhala & Mturi 2008; Molla *et al*. 2008; Tengia-Kessy *et al*. 2010; Ndetei *et al*. 2010; Ao *et al*. 2011; Atwoli *et al*. 2011; Regassa & Kedir 2011). Two-thirds of the studies employed a face-to-face interviewing approach, and a third of studies used self-administered questionnaires; however, all but two studies used self-reported alcohol use. The remaining studies used the alcohol breathalyser (Odero & Zwi 1997; Ayuku & Odero 2002).

**Table 2:** Quality of the papers included in the systematic review and meta-analysis

Quality variable	Quality variable categories	Number of studies	Proportion (%)
Sampling	Non probability	14	25.0
	Probability	42	75.0
Alcohol use information collection	Breathalyser	2	3.6
	Alcohol Use Disorder Identification Test	2	3.6
	CAGE	6	10.7
	Other self-reports	46	82.1
Response rate	Between 60 and 80%	6	10.7
	Above 80%	33	58.9
	Not reported	17	30.4
Interview modality	Face to face	36	64.3
	Self-administered	20	35.7

### Ever use of alcohol

**Figure 2:**
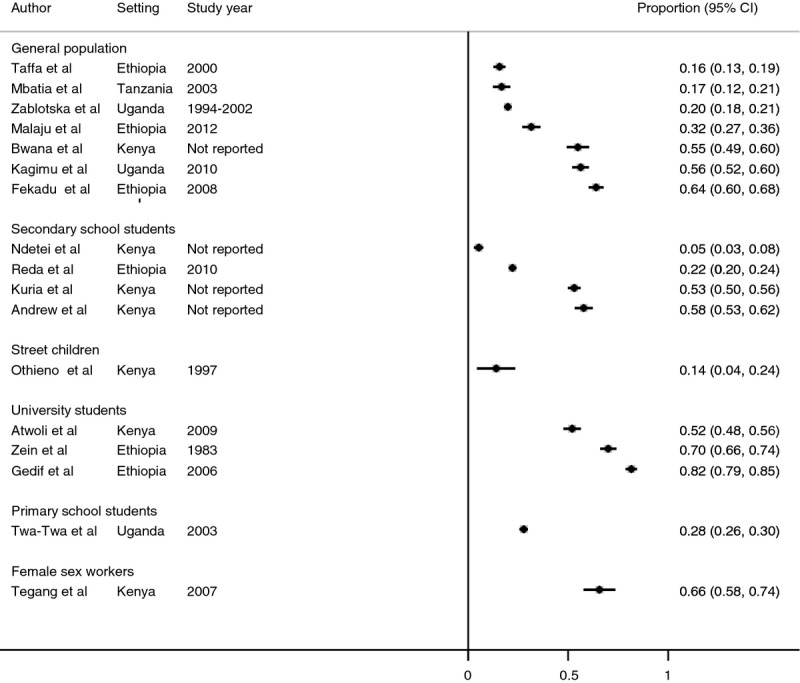
Prevalence of ever use of alcohol among studies included in the systematic review and meta-analysis.

Figure 2 shows the prevalence of reported ever use of alcohol by population groups, including female sex workers, street children, primary school students, secondary school students, general population and university students. Prevalence of reported ever use was highest in the studies among university students [median = 70% interquartile range (IQR): 52–82%] and female sex workers (66%; 95%CI 58–74%). The median prevalence in the four studies among secondary school students was 37% (IQR: 23–56%), although the range was wide, with one study from rural Kenya (Ndetei *et al*. 2010) reporting a prevalence of only 5%. Prevalence was lower among the primary school students (28%; 95%CI: 26–30%), general populations (median = 32%, IQR 17–56%), and among street children 14% (95%CI: 4–24%). Three studies reported gender-specific prevalence of ever-used alcohol; the prevalence was high among females in primary school (36% *vs* 23%) and street children (21% *vs* 11%) and high in male university students (53% *vs* 50%). There was significant heterogeneity based on I^2^ statistics in all subgroups, and therefore, we do not report pooled prevalence.

### Use of alcohol in the past 12 months

Four studies reported the prevalence of alcohol use in the last 12 months. These showed similar levels as found for reported current alcohol use. Three studies were from the general population (median prevalence = 29%, IQR: 21–34%) with a pooled prevalence of 30% (95%CI: 27–33%) and one from university students (prevalence = 22%, 95%CI: 19–25%). One study reported gender-specific prevalence; it was high among males (34% *vs* 17%).

### Current use of alcohol

**Figure 3:**
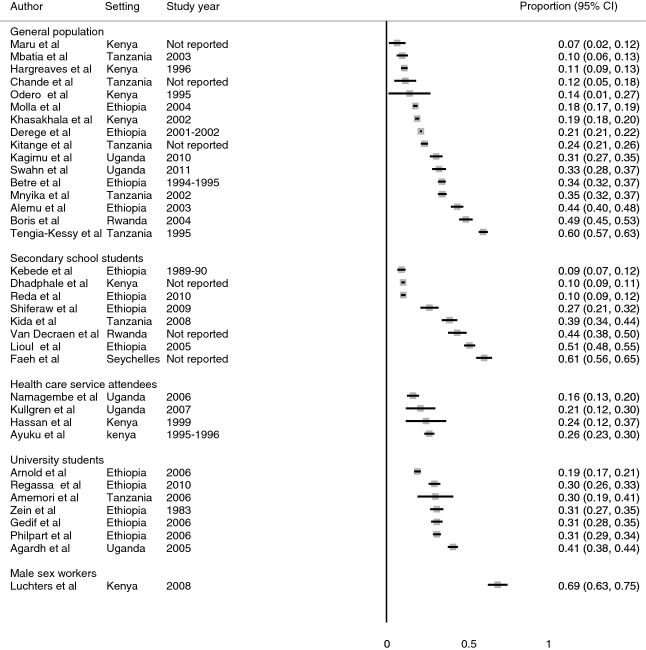
Prevalence of current alcohol use among studies included in the systematic review and meta-analysis.

Current alcohol use may be more relevant than ever use for designing intervention strategies. The prevalence of reported current alcohol use is presented in Figure 3 for general populations, healthcare service attendees, male sex workers, secondary school students and university students. Heterogeneity was highest in studies conducted in general populations and among secondary school students. The prevalence was highest in the one study among male sex workers (69%; 95%CI: 63–75%). Median prevalence among secondary school students was 33% (IQR: 10–48%) and 31% (IQR: 30–31%) among university students and was lower in the general population (median 22%; IQR: 13–34%) and healthcare attenders (median 23%; IQR: 16–24%). Eight studies reported gender-specific prevalences. The median prevalence was high among males; 21% (IQR: 7–26%) *vs*. 9%(IQR: 9–20%) in the general population, 28% (IQR: 13–44%) *vs* 19%(IQR: 7–32%) among healthcare service attenders, 60% (IQR: 56–63%) *vs*. 41% (IQR: 24–58%) in secondary schools, and 43% *vs*. 28% in a university. There was no significant heterogeneity in studies conducted among university students except one study among female university students (Arnold *et al*. 2008). Reported current alcohol use was more common among males than females and not associated with other factors.

### Problem drinking

Problem drinking among young people was reported in five studies, three from the general population, one from female bar workers and one from male sex workers. The prevalence of problem drinking was 36% (95%CI: 33–40%) among female bar workers and 47% (95%CI: 40–53%) among male sex workers. Median prevalence in the general population was 3% (IQR: 1–15%); two of the studies in the general population were from Ethiopia and showed low levels of problem drinking (1–3%), but a study from Tanzania reported a rather high median prevalence of 15% (95%CI: 10–20%).

In this review, eight studies reported problematic drinking, two studies applied AUDIT, and six (13%) used CAGE to screen for problem drinking; two of the studies that applied CAGE and one study that applied AUDIT did not report scores according to age groups (Kullgren *et al*. 2009; Mbatia *et al*. 2009; Namagembe *et al*. 2010).

## Discussion

Among young people in eastern Africa, alcohol use is common and its extent of use varies between specific populations and settings. Due to high level of heterogeneity between studies, we did not report pooled prevalence. The prevalence of reported ever use was highest among university students (70%) and female sex workers (66%) and lower among the general population and primary school students. Few studies reported alcohol use in the last year, and median prevalence in the general population was 29%. Reported current alcohol use was highest among male sex workers (69%), followed by the university students (33%), and was lowest in the general population and secondary school students with the exception of one study in the Seychelles that reported a high prevalence of 61%. Problem drinking was highest among groups known to engage in high-risk behaviours (such as bar workers and sex workers). Generally, reported alcohol use across all definitions of use was highest among groups known to engage in high-risk behaviours, followed by university students. Individuals attending healthcare services and general populations and secondary school students reported the lowest prevalence. Studies included in this review were of good quality; however, about two-thirds employed face-to-face interviewing approaches, an approach prone to social desirability bias that could lead to underreporting of alcohol use.

The varied prevalence of reported alcohol use among groups of young people is potentially due to specific population characteristics (general population *vs*. students *vs*. sex workers). We also attribute these variations to social influence and peer pressure (Smith & Foxcroft 2009; Li *et al*. 2010a,b). For example, the high prevalence of reported alcohol use among secondary school students from the Seychelles may be attributed to the relatively high purchase power of young people in this study population (Faeh *et al*. 2006). In addition, study settings, gender and use of non-standardised alcohol screening questionnaires could partially explain these variations.

Problem drinking was common among young sex workers and female bar workers. Several factors may have contributed to this, including the intertwined nature of bar work and transactional sex – the negotiation of commercial sex usually involves drinking. Also bar workers' psychosocial history, multiple sexual partners, level of education, religion, marital status, number of pregnancies and living conditions, for example, not living within the drinking establishment were found to influence problematic drinking in studies from eastern Africa (Ao *et al*. 2011; Kagimu *et al*. 2013).

The varied prevalence of reported alcohol use for specific groups of young people and the varied risk factors associated with it implies that the need for alcohol interventions is not uniform for all groups of young people. The interventions should address specific needs of a targeted group. For example, in the eastern Africa context, we may need to develop specific strategies to reduce harmful alcohol use among college students, bar workers and commercial sex workers.

An important finding of our review was lack of data on the initiation and persistence of alcohol use among young people in this region. Few studies mentioned factors associated with the initiation of alcohol use, such as peer influence, family and friends, religion and sexual experiences (Otieno & Ofulla 2009; Ndetei *et al*. 2010; Amemori *et al*. 2011; Atwoli *et al*. 2011). Future studies should aim to elicit in-depth information on social factors influencing alcohol use to inform potential interventions.

The use of effective and validated instruments for the screening and assessment of alcohol use is essential to guide research and is important for the design and evaluation of interventions. AUDIT is validated and recommended by WHO for use at the primary healthcare settings and for the assessment of AUD in developing countries (Saunders *et al*. 1993; Chishinga *et al*. 2011; Kapiga *et al*. 2013); it is, however, not widely used for the assessment of AUD among young people in general populations. In our review, only two studies used the internationally recommended AUDIT alcohol screening questionnaire (Mbatia *et al*. 2009; Luchters *et al*. 2011).

## Conclusion

Reported alcohol use among young people in eastern Africa is common and varies between different populations. The prevalence of AUD was highest among populations known to engage in high-risk sexual behaviours, but was also high among students in some of the studies. The studies reviewed lacked data about initiation and persistence of alcohol use, and little information was available about risk factors associated with alcohol use, and AUD. Notably, only few of the studies reviewed used internationally recommended and validated screening questionnaires such as AUDIT. Future epidemiological studies on alcohol use among young people should apply these questionnaires to facilitate comparison. However, such questionnaires have not been evaluated among young people in Africa, and studies closing this knowledge gap are therefore also required. Future studies should also determine factors responsible for initiation, persistence, and patterns of use in preparation for potential interventions. There is an urgent need of targeted interventions for groups of young people with a particularly high risk of alcohol use and AUD such as college students and young sex workers.

## Supporting Information

**Appendix S1.** Search Strategies.
