# Supplementary material for: Systematic review and meta-analysis: prevalence of alcohol use among young people in eastern Africa
Source: Trop Med Int Health. 2014 Jan 31;19(4):476–88. doi: 10.1111/tmi.12267 (PMC4065366; doi:10.1111/tmi.12267)
Supplement: Supplementary file 1 [file tmi0019-0476-SD1.pdf]

**Appendix S1: Search Strategies**

|  | **MEDLINE database** | |
| --- | --- | --- |
|  | **Search terms ( text words and subject headings)** | **Citations retrieved** |
| 1 | exp Alcohol Drinking/ae, ep, mo, pc, px [Adverse Effects, Epidemiology, Mortality, Prevention & Control, Psychology] | 26465 |
| 2 | alcohol abuse.mp. or exp Alcoholism/ | 71593 |
| 3 | alcohol$.mp. | 281211 |
| 4 | exp alcohol-related disorders/ or exp alcohol-induced disorders/ or exp alcoholic intoxication/ or exp alcoholism/ | 95482 |
| 5 | youth$.mp. or exp Adolescent/ | 1580841 |
| 6 | Adolescent/ or teenage$.mp. | 1573769 |
| 7 | (adolescent or child or student).mp. [mp=title, abstract, subject headings, heading word, drug trade name, original title, device manufacturer, drug manufacturer, device trade name, keyword] | 2494555 |
| 8 | persons/ or age groups/ or adolescent/ or young adult/ | 1712711 |
| 9 | africa.mp. or exp Africa/ | 198423 |
| 10 | Tanzania.mp. or exp Tanzania/ | 8583 |
| 11 | Kenya.mp. or exp Kenya/ | 13071 |
| 12 | Somaliland.mp. | 76 |
| 13 | Uganda.mp. or exp Uganda/ | 8859 |
| 14 | Burundi.mp. or exp Burundi/ | 611 |
| 15 | Rwanda.mp. or exp Rwanda/ | 1657 |
| 16 | Eritrea.mp. or exp Eritrea/ | 312 |
| 17 | Ethiopia.mp. or exp Ethiopia/ | 7317 |
| 18 | Djibouti.mp. or exp Djibouti/ | 261 |
| 19 | Somalia.mp. or exp Somalia/ | 1425 |
| 20 | Seychelles.mp. or exp Seychelles/ | 479 |
| 21 | South-Sudan.mp. | 117 |
| 22 | Comoros.mp. or exp Comoros/ | 299 |
| 23 | 1 or 2 or 3 or 4 | 282093 |
| 24 | 5 or 6 or 7 or 8 | 2634394 |
| 25 | 9 or 10 or 11 or 12 or 13 or 14 or 15 or 16 or 17 or 18 or 19 or 20 or 21 or 22 | 202327 |
| 26 | 23 and 24 | 48829 |
| 27 | 25 and 26 | 1235 |

|  | **EMBASE database** | |
| --- | --- | --- |
|  | **Search terms ( text words and subject headings)** | **Citations retrieved** |
| 1 | exp drinking behavior/co, ep [Complication, Epidemiology] | 3267 |
| 2 | alcoholism.mp. or exp alcoholism/ | 113395 |
| 3 | exp alcohol abuse/ | 20973 |
| 4 | alcohol drinking.mp. or exp drinking behavior/ | 36967 |
| 5 | alcohol/ or alcohol consumption/ or alcoholism/ | 307569 |
| 6 | alcohol consumption/ or drunk$.mp. or alcohol/ or alcoholism/ or drunkenness/ or alcohol intoxication/ | 315047 |
| 7 | (adolescent or child or student).mp. [mp=title, abstract, subject headings, heading word, drug trade name, original title, device manufacturer, drug manufacturer, device trade name, keyword] | 2652116 |
| 8 | exp juvenile/ or adolescent/ | 1263951 |
| 9 | youth$.mp. | 47218 |
| 10 | (young adj2 people).mp. [mp=title, abstract, subject headings, heading word, drug trade name, original title, device manufacturer, drug manufacturer, device trade name, keyword] | 21808 |
| 11 | (early adj2 adult$).mp. [mp=title, abstract, subject headings, heading word, drug trade name, original title, device manufacturer, drug manufacturer, device trade name, keyword] | 6646 |
| 12 | (young adj2 adult$).mp. [mp=title, abstract, subject headings, heading word, drug trade name, original title, device manufacturer, drug manufacturer, device trade name, keyword] | 75907 |
| 13 | teenage$.mp. or exp adolescence/ | 86727 |
| 14 | exp Africa/ or africa.mp. | 239298 |
| 15 | Tanzania.mp. or exp Tanzania/ | 10057 |
| 16 | Kenya.mp. or exp Kenya/ | 14834 |
| 17 | Somaliland.mp. | 150 |
| 18 | Uganda.mp. or exp Uganda/ | 11677 |
| 19 | Burundi.mp. or exp Burundi/ | 701 |
| 20 | Rwanda.mp. or exp Rwanda/ | 1940 |
| 21 | Eritrea.mp. or exp Eritrea/ | 455 |
| 22 | Ethiopia.mp. or exp Ethiopia/ | 8375 |
| 23 | Djibouti.mp. or exp Djibouti/ | 342 |
| 24 | Somalia.mp. or exp Somalia/ | 1617 |
| 25 | Seychelles.mp. or exp Seychelles/ | 566 |
| 26 | South-Sudan.mp. | 150 |
| 27 | Comoros.mp. or exp Comoros/ | 309 |
| 28 | 1 or 2 or 4 or 5 or 6 | 344799 |
| 29 | 7 or 8 or 9 or 10 or 11 or 12 or 13 | 2748730 |
| 30 | 14 or 15 or 16 or 17 or 18 or 19 or 20 or 21 or 22 or 23 or 24 or 25 or 26 or 27 | 244768 |
| 31 | 28 and 29 | 42424 |
| 32 | 30 and 31 | 835 |

|  | **PsycINFO database** | |
| --- | --- | --- |
|  | **Search terms ( text words and subject headings)** | **Citations retrieved** |
| 1 | exp drinking behavior/ [Complication, Epidemiology] | 55829 |
| 2 | alcoholism.mp. or exp alcoholism/ | 32794 |
| 3 | exp alcohol abuse/ | 37188 |
| 4 | alcohol drinking.mp. or exp drinking behavior/ | 56991 |
| 5 | alcohol/ or alcohol consumption/ or alcoholism/ | 24035 |
| 6 | alcohol consumption/ or drunk$.mp. or alcohol/ or alcoholism/ or drunkenness/ or alcohol intoxication/ | 28229 |
| 7 | (adolescent or child or student).mp. [mp=title, abstract, subject headings, heading word, drug trade name, original title, device manufacturer, drug manufacturer, device trade name, keyword] | 444202 |
| 8 | youth$.mp. | 60262 |
| 9 | (young adj2 people).mp. [mp=title, abstract, subject headings, heading word, drug trade name, original title, device manufacturer, drug manufacturer, device trade name, keyword] | 16285 |
| 10 | (early adj2 adult$).mp. [mp=title, abstract, subject headings, heading word, drug trade name, original title, device manufacturer, drug manufacturer, device trade name, keyword] | 3689 |
| 11 | (young adj2 adult$).mp. [mp=title, abstract, subject headings, heading word, drug trade name, original title, device manufacturer, drug manufacturer, device trade name, keyword] | 29706 |
| 12 | teenage$.mp. | 10139 |
| 13 | exp Africa/ or africa.mp. | 13495 |
| 14 | Tanzania.mp. or exp Tanzania/ | 1127 |
| 15 | Kenya.mp. or exp Kenya/ | 1798 |
| 16 | Somaliland.mp. | 11 |
| 17 | Uganda.mp. or exp Uganda/ | 1455 |
| 18 | Burundi.mp. or exp Burundi/ | 69 |
| 19 | Rwanda.mp. or exp Rwanda/ | 479 |
| 20 | Eritrea.mp. or exp Eritrea/ | 76 |
| 21 | Ethiopia.mp. or exp Ethiopia/ | 758 |
| 22 | Djibouti.mp. or exp Djibouti/ | 10 |
| 23 | Somalia.mp. or exp Somalia/ | 214 |
| 24 | Seychelles.mp. or exp Seychelles/ | 36 |
| 25 | South-Sudan.mp. | 16 |
| 26 | Comoros.mp. or exp Comoros/ | 5 |
| 27 | 1 or 2 or 3 or 5 or 6 | 63552 |
| 28 | 7 or 8 or 9 or 10 or 11 or 12 | 507329 |
| 29 | 13 or 14 or 15 or 16 or 17 or 18 or 19 or 20 or 21 or 22 or 23 or 24 or 25 or 26 | 18024 |
| 30 | 27 and 28 | 10307 |
| 31 | 29 and 30 | 49 |

|  | **GLOBAL HEALTH database** | |
| --- | --- | --- |
|  | **Search terms ( text words and subject headings)** | **Citations retrieved** |
| 1 | drinking behavior.mp. or alcoholism.sh. or behaviour.sh. or alcohol intake.sh. or alcoholic beverages.sh. | 64697 |
| 2 | exp alcoholism/ or addiction/ or alcoholic beverages/ | 14438 |
| 3 | alcohol abuse.mp. or drug users.sh. or alcohol intake.sh. or alcoholism.sh. or substance abuse.sh. or drug abuse.sh. or alcoholic beverages.sh. | 32358 |
| 4 | alcohol drinking.mp. or alcohol intake.sh. or alcoholic beverages.sh. or drinking.sh. or alcohols.sh. or alcoholism.sh. | 27779 |
| 5 | alcohol/ or alcohol consumption/ or alcoholism/ | 19164 |
| 6 | alcohol consumption/ or drunk$.mp. or alcohol/ or alcoholism/ or drunkenness/ or alcohol intoxication/ | 20623 |
| 7 | alcohol consumption.mp. or exp alcohol intake/ | 17689 |
| 8 | exp adolescents/ or school children/ or young adults/ | 53213 |
| 9 | (adolescent or child or student).mp. [mp=abstract, title, original title, broad terms, heading words] | 79179 |
| 10 | juvenile .mp. or adolescent.mp. | 18875 |
| 11 | youth$.mp. or exp youth/ | 13275 |
| 12 | (young adj2 people).mp. [mp=title, abstract, subject headings, heading word, drug trade name, original title, device manufacturer, drug manufacturer, device trade name, keyword] | 4919 |
| 13 | (early adj2 adult$).mp. [mp=title, abstract, subject headings, heading word, drug trade name, original title, device manufacturer, drug manufacturer, device trade name, keyword] | 1117 |
| 14 | (young adj2 adult$).mp. [mp=abstract, title, original title, broad terms, heading words] | 15404 |
| 15 | teenage$.mp. or exp adolescence/ | 33844 |
| 16 | exp Africa/ or africa.mp. | 177386 |
| 17 | Tanzania.mp. or exp Tanzania/ | 9516 |
| 18 | Kenya.mp. or exp Kenya/ | 12567 |
| 19 | Somaliland.mp. | 352 |
| 20 | Uganda.mp. or exp Uganda/ | 8962 |
| 21 | Burundi.mp. or exp Burundi/ | 601 |
| 22 | Rwanda.mp. or exp Rwanda/ | 1318 |
| 23 | Eritrea.mp. or exp Eritrea/ | 573 |
| 24 | Ethiopia.mp. or exp Ethiopia/ | 6801 |
| 25 | Djibouti.mp. or exp Djibouti/ | 300 |
| 26 | Somalia.mp. or exp Somalia/ | 1458 |
| 27 | Seychelles.mp. or exp Seychelles/ | 295 |
| 28 | South-Sudan.mp. | 117 |
| 29 | Comoros.mp. or exp Comoros/ | 267 |
| 30 | 1 or 2 or 3 or 4 or 5 or 6 or 7 | 80444 |
| 31 | 8 or 9 or 10 or 11 or 12 or 13 or 14 or 15 | 138201 |
| 32 | 16 or 17 or 18 or 19 or 20 or 21 or 22 or 23 or 24 or 25 or 26 or 27 or 28 or 29 | 178733 |
| 33 | 30 and 31 | 13508 |
| 34 | 32 and 33 | 920 |

|  | **AFRICAWIDE INFORMATION database** | |
| --- | --- | --- |
|  | **Search terms ( text words and subject headings)** | **Citations retrieved** |
| 1 | alcohol-drinking | 1599 |
| 2 | alcohol-abuse | 2469 |
| 3 | drug-abuse | 3036 |
| 4 | alcoholism* | 9922 |
| 5 | alcohol-consumption | 1580 |
| 6 | Alcohol and college | 528 |
| 7 | Drug addiction | 2789 |
| 8 | Adolescent | 111166 |
| 9 | Students | 40504 |
| 10 | Young-adults | 26100 |
| 11 | young-people | 5087 |
| 12 | alcohol-beverages | 21 |
| 13 | alcoholic | 3730 |
| 14 | youths | 28174 |
| 15 | young N2 people | 5245 |
| 16 | young N2 adults | 26604 |
| 17 | Africa | 1693539 |
| 18 | Tanzania | 41493 |
| 19 | Kenya | 79650 |
| 20 | Somaliland | 2355 |
| 21 | Uganda | 48702 |
| 22 | Burundi | 15167 |
| 23 | Rwanda | 17680 |
| 24 | Eritrea | 6647 |
| 25 | Ethiopia | 42965 |
| 26 | Djibouti | 4111 |
| 27 | Somalia | 23560 |
| 28 | Seychelles | 3199 |
| 29 | South-Sudan | 2354 |
| 30 | Comoros | 2685 |
| 31 | 1 or 2 or 3 or 4 or 5 or 6 or7 or 12 or 13 | 18298 |
| 32 | 8 or 9 or10 or11 or14 or 15 or 16 | 184589 |
| 33 | 17 or 18 or 19 or 20 or 21 or 22 or24 or 25 or 26 or 27 or 28 or 29 or 30 | 1780419 |
| 34 | 31 and 32 and 33 | 974 |
